# COVID-19 and human resources for health: analysis of planning, policy responses and actions in Latin American and Caribbean countries

**DOI:** 10.1186/s12960-023-00795-8

**Published:** 2023-03-14

**Authors:** Juana Paola Bustamante Izquierdo, E. Benjamín Puertas, Diana Hernández Hernández, Hernán Sepúlveda

**Affiliations:** 1 Health Labour Market Unit, Health Workforce Department, Universal Health Coverage Cluster, World Health Organisation (WHO/UHC/HWF), 1211 Geneva, Switzerland; 2 Human Resources for Health for the Sub-Regional Programme for the Caribbean, Human Resources for Health Unit, Health Systems and Services Area, Office of the Assistant Director (PAHO/AD/HSS/HR), Pan American Health (PAHO/WHO), Washington, United States of America; 3 Health Labour Market Unit, WHO (WHO/UHC/HWF), Geneva, Switzerland; 4 Human Resources for Health for the Sub-Regional Programme for South America, Human Resources for Health Unit (PAHO/AD/HSS/HR), Washington, United States of America

**Keywords:** Health workers, Health Labour Market, COVID-19, Emergency preparedness, Working conditions, Supply, Demand

## Abstract

**Background:**

The COVID-19 pandemic led to worldwide health service disruptions, due mainly to insufficient staff availability. To gain insight into policy responses and engage with policy-makers, the World Health Organization (WHO) developed a global approach to assess and measure the impact of COVID-19 on the health workforce. As part of this, WHO, together with the Pan American Health Organization (PAHO), supported an impact analysis of COVID-19 on health workers and policy responses, through country case studies in Latin America and the Caribbean (LAC).

**Methods:**

We sought to identify lessons learned from policies on human resources for health (HRH) during health emergencies, to improve HRH readiness. First, we performed a rapid literature review for information-gathering. Second, we used the WHO interim guidance and impact measurement framework for COVID-19 and HRH to systematically organize that information. Finally, we used the Health Labour Market Framework to guide the content analysis on COVID-19 response in eight LAC countries and identify lessons learned to improve HRH readiness.

**Results:**

Planning and implementing the COVID-19 response required strengthening HRH governance and HRH data and information systems. The results suggest two main aspects for HRH governance crucial to enabling an agile response: (1) aligning objectives among ministries to define and produce regulation and policy actions; and (2) agreeing on the strategy for HRH management between the public and private sectors, and between central and local governments. We identified three areas for improvement: (a) HRH information systems; (b) methodologies to estimate HRH needs; and (c) teams to analyse information for decision-making. Three key actions were identified during countries monitored, reviewed, and updated their response stages: (i) strengthening response through primary health care; (ii); planning HRH needs to implement the vaccination plan; and (iii) securing long-term HRH availability.

**Conclusion:**

Countries coordinated and articulated with different stakeholders to align objectives, allocate resources, and agree on policy actions to implement the COVID-19 response. Data and information for HRH preparedness and implementation were key in enabling an agile COVID-19 response and are key areas to explore for improved pandemic preparedness.

## Introduction

The COVID-19 pandemic has strongly impacted population health and put pressure on the entire health system^1^ [1]. It led to health service disruptions around the world, due mainly to insufficient staff availability^2^ [2]. COVID-19 has exposed health workers to infection, fatigue, occupational burnout, stress, harassment and physical and psychological violence. Efforts have been made to assess the pandemic’s direct impact on human resources for health (HRH). However, global estimates of COVID-19 infections and deaths among HRH tend to suggest that official reporting mechanisms do not capture the full scale of this impact [3].^3^

The pandemic has emphasized such HRH concerns as shortage and maldistribution as well as inadequate payment and decent working conditions, e.g., lack of contract stability. This added strain to the health system deepened any existing staff unavailability, affecting both teamwork and health workers’ mental health.^4^ Additionally, it has highlighted insufficiencies vis-à-vis data and information systems, while demonstrating the importance of data science in planning and reviewing the COVID-19 response related to HRH issues. However, the COVID-19 pandemic also has led to identifying ways to rapidly recruit, train, and protect the health workforce. This has led countries to use various mechanisms to plan and respond to COVID-19 from the HRH perspective [4–6] .

To address HRH-related COVID-19 challenges, the World Health Organization (WHO) developed a global approach to better assess and measure the impact of COVID-19 on HRH, to gain insight into management and policy responses as well as engagement with policy-makers. Thus, WHO developed an interim guidance on HRH policy and management in the context of the COVID-19 pandemic response [7] as well as a standardized impact measurement framework [8]. Additionally, living systematic reviews were undertaken, aimed at gathering information and analytics on health and care workers in the context of COVID-19. Alongside this process, health workforce intelligence related to COVID-19 was collected from open sources to complement the reviews. All the information collected on COVID-19 and HRH resulted in a World Health Assembly resolution, namely the *Global health and care worker compact* [9], which provides recommendations on how to protect health workers, safeguard their rights, and promote and ensure decent work. The overall aim was to support policy dialogue and advocacy opportunities.

This paper constitutes an effort to build on the International Year of Health and Care Workers (2021) and the strategic objectives of the *Global strategy on human resources for health* [10], aiming to strengthen support to countries as they design and implement strategies addressing health workers’ problems during COVID-19. Thus, WHO—together with two of its Regional Offices, the Pan American Health Organization (PAHO) and the WHO Regional Office for Africa (AFRO)—have supported COVID-19 impact analyses regarding health workers and policy responses by developing case studies from selected countries. These use a standardized methodology based on WHO interim guidance [7], the standardized impact measurement framework [8], and the Health Labour Market Framework [10, 11].

Gaining better insight into the policy response to COVID-19 is crucial for addressing the challenges ensuing from COVID-19. Nonetheless, as countries attempt to do so, there remains a lack of systematic knowledge on mechanisms and policies adopted by countries from different geographic areas and institutional governance to address HRH challenges. This paper is aimed at identifying lessons learned on HRH to better address health emergencies and build improved post-pandemic health systems. We do this through a secondary analysis of findings from the two sub-regional reports on eight countries of Latin America and the Caribbean.

The paper is organized as follows: the first section summarizes the methods used to develop the analysis. The second presents the mechanisms and policy responses adopted during the COVID-19 pandemic response, as analysed across the preparedness, implementation, and monitoring/updating phases. The final section presents lessons learned and concluding remarks.

## Method

We adopted a multi-pronged approach for this paper. First, we conducted a rapid literature review on the background material for the case studies. Second, to systematically organize that information, we used the WHO interim guidance [7] and impact measurement framework for COVID-19 and HRH [8]. Finally, we used the HLM framework to analyse the information [11].

We carried out our literature review in 2020 and 2021, focusing on reports, grey literature and desk review of policies adopted during the pandemic, obtained from ministries of health (MoHs) and service delivery agencies; review of health information systems (HIS) and national human resource information systems (HRISs); and surveillance databases for data on HRH infections. The details have been published in the *The impact of COVID-19 on human resources for health and policy response: the case of Plurinational State of Bolivia, Chile, Colombia, Ecuador and Peru* [12].^5^ The present study adds three Caribbean countries (Belize, Grenada and Jamaica) [13].^6^ The data and information reported spans the period between March 2020 and April 2021. In this study, we focused on countries from the PAHO Region (i.e. the Americas) where the reports and background material on the selected countries^7^ systematically followed the WHO interim guidance [7]. Additionally, the countries participating in the reports are grouped into sub-regions developing common HRH-related policies where they are also part of sub-regional organizations like the Andean Health Agency/Hipólito Unanue Agreement *(Organismo Andino de Salud–Acuerdo Hipólito Unanue)* (ORAS-CONHU) and the Caribbean Community (CARICOM).

We used the domains listed in the guidelines from the WHO interim guidance [7], as well as the multidimensional factors affecting HRH listed in the standardized impact measurement framework [8]. This enabled us to organize the information on HRH policy response during COVID-19 in the eight Latin American and Caribbean countries around the three response pillars supporting health emergencies [14]: 1 workforce readiness in preparedness, 2 implementation; and 3 monitoring/updating the response.

We used the HLM framework to carry out the content analysis of the background documents and information on HRH response. This enabled us to understand mismatches and market failures in the health labour market (HLM) and thus identify and define policies and actions to build HRH readiness that currently guide the discussion on policy dialogue. For the Caribbean subregion, we achieved this in the HRH task force sessions; and for the Andean countries, through meetings and webinars held between 2021 and 2022. The outcome of these discussions led to policy briefs and identification of key elements strengthening HRH. We integrated the information into this paper in the form of challenges and actions identified to improve HRH in different areas of the three pillars to support improved response during health emergencies.

## HRH in the context of the response to health emergencies

In this section, we discuss key HRH strategies used by countries during the COVID-19 policy response and connect them to each pillar supporting health emergencies:

1. Workforce readiness for initial response: We discuss the importance of having and analysing HRH data to identify both the need for and availability of HRH to support response.
2. Implementation while strengthening HRH: We highlight the importance of HRH governance during the pandemic for implementing measures to increase, maintain and protect health workers. We also summarize these measures.
3. Continually monitoring, reviewing and updating the response to keep responding to new waves of COVID-19 and the challenge of vaccination.

### Workforce readiness for initial response

Data on HRH and information analysis are key elements in planning preparedness to respond to a health emergency. We identify three mechanisms for improving real-time, comprehensive and detailed information for dialogue and decision-making when preparing for health emergencies: (a) HRH information systems (HRIS); (b) methodologies to estimate HRH needs; and (c) teams to analyse information for decision-making. Table 1 shows the eight countries of the case studies classified under this schema.

**Table 1:** HRH information for workforce readiness in preparedness for the COVID-19 response

Mechanism	Stage of development		
	Mechanism not or only initially developed^a^	Mechanism in place but not used for policy decisions^b^	Mechanism in place and used for policy dialogue/decisions^c^
HRH Information System (HRIS)	Belize^d^ Bolivia ^e^ Grenada^d^ Jamaica^f^		Chile Colombia Ecuador Peru
Methodologies for estimating HRH gap	Bolivia^g^ Colombia^g^ Grenada^h^ Jamaica^h^	Belize^i^	Chile^k^ Ecuador^j^ Peru^j^
HRH planning teams	Bolivia^l^	Belize^m^ Grenada^m^ Jamaica^m^	Chile^n^ Colombia^n^ Ecuador^n^ Peru^n^

Elaboration based on PAHO [13] and WHO [12]

^a^Countries that have not developed or are in process of creating this mechanism of HRH information

^b^Countries that have already created this mechanism of HRH information but do not use the mechanisms to support decision-making

^c^Countries used the HRH information mechanism to support decision-making

^d^Countries that collect information for a few occupational profiles but do not report it on a regular basis

^e^Countries that collect information through their Health Entities Single Registry (HESR in English, *Registro Único de Establecimiento de Salud* [RUES] in Spanish) but do not report it on a regular basis

^f^Countries that report information on a few occupational profiles on a regular basis but have not developed a HRH information system

^g^There was no evidence of this before the pandemic. During COVID-19, the country used the PAHO model to estimate supply of and demand of HRH

^h^Countries that conducted capacity-building on the methodology for Workload Indicators of Staffing Need (WISN)

^i^The WISN methodology was used for planning and mobilizing healthcare workers for COVID-19, but there is no evidence of the need to quantify HRH

^j^Countries where the supply and demand estimation method was developed before the pandemic

^k^The MoH-Chile periodically estimates the HRH deficit to negotiate the budget needed for addressing the HRH shortage, using a methodology known and approved by Chilean Ministry of Finance

^l^There was no team in Bolivia before de pandemic. However, one was created as part of strengthening the MoH-Bolivia

^m^There is a unit dedicated to HRH management, but there is no evidence relevant to HRH information analysis purposes

^n^There is a team dedicated to analysing HRH information

HRH information differed in the countries studied, both by frequency of collection and by occupational groups. Data reported in WHO’s National Health Workforce Accounts (NHWA),^8^ such as stock or density by occupation [15], show that in Chile, Colombia, Ecuador, Peru and Jamaica, information on HRH is collected regularly on an annual basis. Belize, however, has data series only on medical doctors and nursing personnel. In contrast, Bolivia and Grenada have data for specific years only on medical doctors, nursing personnel, dentists, and pharmacists [15]. Thus, challenges remain, such as standardizing indicators on HRH and improving HRIS interoperability.

Table 2 outlines the HRIS in six LAC countries, both before and after the COVID-19 pandemic, to track confirmed COVID-19 cases and deaths among HRH. The data published vary in terms of access of information and whether publicly available or not; frequency of publication (regular or non-regular basis); and disaggregation of data by occupational group, sub-national level, sex, age and source of infection. Collecting and analysing information on total HRH and COVID-19 confirmed cases and deaths therein contributed to strengthening existing HRH data and information analysis.

**Table 2:** HRIS available in the countries studied before and during the COVID-19 pandemic

Country	Before pandemic	During pandemic
Bolivia	Professional Registration System *(Sistema de Registro Profesional*/SiREPRO) HRH module of Single Registry of Health Establishments *(Registro Único de Establecimientos de Salud*/RUES)	Created during pandemic: Integrated Epidemiological Surveillance System for COVID (COVID SIVE*/Sistema Nacional de Vigilancia Epidemiológica)* It is not publicly available
Chile	Human Resources Information System (SIRH/*Sistema de Información de Recursos Humanos)* Individual Provider Registry *(Registro de Proveedores Individuales)*	Epidemiological Surveillance System *(Sistema de Vigilancia Epidemiológica*/EPIVIGILA Publication of two specific journals on HRH
Colombia	National Registry of Healthcare Talent *(Registro Único Nacional del Talento Humano en Salud /* ReTHUS)	Public Health Surveillance System *(Sistema Nacional de Vigilancia en Salud Pública*/SIVIGILA) Enrolment in ReTHUS increased considerably so health workers could receive the COVID-19 bonus Weekly journals in the web page of the National Health Institute *(Instituto Nacional de Salud /* INS)
Ecuador	System to register medical qualifications to ensure quality of care *(Agencia de Aseguramiento de la Calidad de los Servicios de Salud y Medicina Prepagada/ACESS)* Statistical Register of Health Resources and Activities *(Registro Estadístico de Recursos y Actividades de Salud*/RAS)	National Epidemiological Surveillance Online System *(Sistema Nacional de Vigilancia en Salud Pública*/VIEPI) Daily mortality and morbidity updates available through the web portal www.coronavirusecuador.com
Peru	National Registry of Health Personnel online system *(Registro Nacional de Personal de la Salud*/INFORHUS)	National Epidemiological Surveillance System (NotiWeb) INFORHUS strengthened to give timely, updated and strategic HRH information to the Ministry of Health and regional governments to aid in decision-making COVID-19 virtual situation room for health-sector workers
Belize	No evidence	Ministry of Health (MoH) Facebook page

Own elaboration based on PAHO [16], WHO [12] and PAHO (forthcoming, [17]

No evidence was found in Grenada and Jamaica

In the five Latin American countries studied, estimations were done on HRH shortages, in order to plan their response to the COVID-19 pandemic. However, we did not find evidence of these calculations in the three Caribbean countries studied. In all five Latin American countries, an initial deficit was projected of 34 261 additional health workers, which represented between 0.54% and 4.17% of total HRH [12]. By occupational group, all five countries had a shortage of doctors and nurses. These shortages led the countries to develop regulations facilitating the hiring and deployment of additional personnel, as well as redeployment of existing personnel. Apart from Colombia, these estimates only applied to the public health sector [12]. Estimating and analysing HRH needs and demand during COVID-19 was useful for identifying total HRH availability in the countries. Doing so also provided evidence to plan the COVID-19 response, in terms of addressing HRH gaps at national and sub-national levels, planning the vaccination strategy and identifying potential financial needs related to HRH (Table 3).

**Table 3:** Methodologies to estimate HRH needs in Latin America and teams dedicated to analysing HRH information

Country	Before pandemic	During pandemic
Bolivia	No evidence of methodologies or teams	Methodologies: HRH supply and needs methodology provided by PAHO Information on HRH payroll enabled understanding of total employment in the health sector by occupational profile Estimations on HRH needs included assumptions on COVID-19 cases involving 50% of HRH in primary health care. However, COVID-19 cases exceeded assumptions Teams: HRH Management and Administration Unit created during the pandemic No evidence on HRH data analysis
Chile	Methodologies: HRH planning methodologies with focus on needs These methodologies have been used on an annual basis to establish the required budget to reduce the existing gap Teams: HRH Information Management Unit	Methodologies: Adaptation of the HRH planning methodology to support informed decision-making Assumptions: HRH needed to treat COVID-19 based on increased number of beds in intensive care units (ICUs) and hospital admissions
Colombia	Methodologies: A report on HRH estimates on supply and needs Teams: Observatory of Human Talent in Health	Methodologies: Supply estimation based on National Registry of Healthcare Talent (ReTHUS) and on number of graduates. Needs calculated by case projection. Support provided for decision-making HRH needed to treat COVID-19 cases estimated, based on increased number of intensive care units (ICUs) and intensive treatment units (ITUs) and beds for them, as well as hospital admissions HRH to treat COVID-19 cases corresponding to general practitioners and specialists, nurses, and therapists Estimations showed shortages of specialists in intensive care (adult and pediatric), internal medicine, anesthesiology and surgery for treatment of COVID-19
Ecuador	Methodologies: Human talent planning for health facilities methodology Teams: National Directorate of Administration of Human Talent *(Dirección de Administración del Talento Humano)* within the Ministry of Public Health	Methodologies**:** Independent methodologies used by the Ministry of Public Health and the Ecuadorian Social Security Institute *(Instituto Ecuatoriano de Seguro Social*/IESS) No evidence on assumptions made
Peru	Methodologies: HRH Supply and Needs Methodology Teams**:** Observatory of Human Resources in Health *(Observatorio de Recursos Humanos en Salud*/ORHUS)	Methodologies: HRH Supply and Needs Methodology Initial estimation of number of health professionals and non-clinical staff required for 3 months Decrease of HRH in the public sector within risk groups

Own elaboration based on WHO [12] and forthcoming PAHO [17]

These projections used methodologies already in place, with additional assumptions in the case of Chile and Colombia. Bolivia used the PAHO model. Peru and Ecuador implemented new methodologies. Teams dedicated to HRH analysis and planning within the ministries of health (MOHs) were key in carrying out analyses to aid in informed decision-making [12] (Table 3).

Through the process of planning the response, countries have identified the importance of both HRH data and information analysis in understanding HRH dynamics and issues, and as well as in identifying policy actions. Policy dialogues in the Region help countries share and discuss their experiences, including ways to improve HRIS interoperability and use evidence to support decision-making vis-à-vis HRH. In the countries studied, this has been key to initiating and/or strengthening the policy-making process addressing workforce readiness.

### Implementation of the pandemic response plan by strengthening HRH

Countries faced the COVID-19 pandemic with pre-existing HRH shortages in key occupational groups, gaps in skills and competencies and/or sub-national HRH imbalances. We identified two aspects of governance^9^ crucial to preparedness and response during the pandemic: (a) aligning HRH objectives among ministries to define and produce regulation and policy actions; and (b) agreeing on the strategy for HRH management between the public and private sectors as well as central and local governments.

Caribbean countries coordinated between the ministries of health and finance to find ways to hire additional HRH [13]. The Latin American countries adopted a coordinated approach among ministries of health, education, labour, and finance to implement mechanisms aimed at increasing HRH availability, as well as their protection and training [12].

Coordination between central and local governments was needed to agree on HRH strategies at sub-national level and to share information. For example, Bolivia, Chile, Colombia and Ecuador fostered central–local relations to implement coordinated mechanisms addressing the HRH deficit [12].

Funding the additional cost of the COVID-19 response was a challenge faced by every country in the study. Aligning the objectives and coordinating subsequent measures were key factors to facilitate the implementation of policy actions and the shifting and/or allocation of financial resources. Belize, Chile, Colombia, Grenada, Jamaica and Peru mainly used existing resources from the general government budget. Ecuador supplemented them with grants and loans from multilateral organizations. In Bolivia, the main funding source was a World Bank loan that had been made available before the pandemic and was repurposed. All countries additionally received donations from other countries and international agencies like PAHO, as well as PAHO technical cooperation. The three Caribbean countries received training, logistics and financial support from PAHO [12, 13].

The eight countries used different mechanisms for coordinating actions with the private sector. In the Caribbean, the private sector was mainly involved through donations to protect and support HRH [13]. In Latin America, there were agreements between the private sector and the government to increase HRH availability (i.e. universities), to provide training and to protect HRH mental health. These involved input and assistance from academic and research entities as well as professional organizations (i.e. licensing boards). Ecuador and Bolivia obtained loans from multilateral organizations to help fund their COVID-19 response [12].

All countries adopted measures to increase or maintain HRH and to protect and support them. These we shall summarize in the next section.

#### Securing and increasing HRH availability

In the Caribbean countries, HRH shortages existed as a direct result of HRH migration [18, 19], especially of nurses. An additional cause was limited HRH production within the countries analysed, thus impacting the quality of care delivery [16]. In line with the *WHO global code of practice on the international recruitment of health personnel* (WHO, 2010), in view of the private sector actively recruiting health workers from countries facing critical health worker shortages, there is a need for enhanced technical, financial and other support to the countries of origin of migrant health workers. Indeed, in order to prevent this from happening, there must be a push to avoid and prevent this practice.

In the Latin American countries, HRH availability dropped when workers had to go into isolation, became ill or died from COVID-19. In countries like Bolivia and Ecuador, health workers refused to attend COVID-19 cases due to lack of PPE and unstable contracts [12]. Table 4 presents eight mechanisms identified across the countries studied to maintain and increase HRH availability during the COVID-19 response. Table 5 presents the strengths and challenges of said mechanisms.

**Table 4:** Measures to maintain and increase HRH availability

Mechanism	Bolivia	Chile	Colombia	Ecuador	Peru	Belize	Grenada	Jamaica
Recruitment of additional HRH	6777 HRH**	19 027 HRH	Recruitment of scarce HRH	3087 HRH***	44 207 HRH***	230 community HRH	118 HRH	1000 community HRH
Temporary reassignment of HRH to COVID-19 treatment	3 600 HRH**	44 of essential non-COVID-19 interventions postponed^****^	78 300 HRH*	10 000 HRH*	80 202 HRH*	Not quantified	Not quantified	Not quantified
Deployment of students and recent and overseas graduates	520 students and recent graduates contracted (220) and trainee students incorporated (300)** Recruitment of HRH holding an unvalidated overseas medical qualifications	Students in their final semesters recruited	1523 students early graduated in general medicine (1328) and specialist medical disciplines (195)* Accelerated validation of foreign qualifications	Cooperation agreements with higher education institutions	Rotation of residents Early completion of residency Temporary authorization to practice without validation of qualifications	Final-year nursing students	Final-year nursing students	X
Work shift changes	Increased working hours for available HRH, even double shifts	Expansion and adjustment	X	X	11 662 HRH worked overtime*	12-h shifts instead of 8-h shifts	12-h shifts instead of 8-h shifts	X
HRH from international cooperation	X	X	150 HRH recruited through UNHCR and USAID*	X	92 HRH from Cuba and Médecins sans Frontières*	121 HRH from Cuba	8 HRH from Cuba	140 HRH from Cuba
Volunteers	450 recent graduates through agreements with universities	1000 students recruited and trained by the University of Chile	X	X	X	500 volunteers	59 volunteers	1000 volunteers
Armed forces	X	HRH to treat COVID-19 patients	Retirees from the armed forces and national police	1523 HRH to strengthen the public network**	X	X	X	X
Retirees	X	1500 professionals released from duty re-entered medical practice	Retired HRH under 60	X	X	X	X	X

Elaboration based on PAHO [16], WHO [12], and Köppen et al. [20]

*August 2020 **October 2020; ***December 2020; ****With reference to 2019

**Table 5:** Strengths and challenges of measures to maintain and increase HRH

Mechanism	Strengths	Challenges
Recruitment of additional HRH	Novel hiring mechanisms or adjustment of existing mechanisms which are susceptible to being replicated	Mechanisms to absorb newly recruited HRH, which depends on identifying adequate sources of funding Balancing decent work with flexibility in hiring and working conditions
Temporary reassignment of HRH to COVID-19 treatment	Allows for optimizing existing supply of HRH	Requires quick training in new diseases treatment specific skills
Deployment of students and recent and overseas graduates	Facilitates the continuous flow of HRH to the neediest areas Mechanisms to relax requirements and accelerate procedures to allow students to graduate so they can enter the workforce	Need for quick training in treating new diseases Challenge in retaining students when once mandatory service ends Mitigation of displacement of national HRH, thus avoiding discrimination Temporary elimination of quality controls
Work shift changes	Allows for optimizing existing supply of HRH	Risk of burnout and mental health impacts
HRH from international cooperation	Facilitates HRH availability lacking in the country	Increase recruitment of international HRH to fill vacancies, usually educated in low- to middle-income countries
Volunteers	HRH availability increased at low or no additional cost Volunteers may include people who had no healthcare training to provide logistical support in general areas in response to the pandemic, allowing for increasing the scope of medical teams	No payment and lack of decent working conditions

Elaboration based on PAHO [16], WHO [12] and Abdul Rahim et al. [21]

#### Protecting and safeguarding HRH

The health emergency acts ratified in the countries allowed for adopting integrated, multidimensional measures to reduce the risk of infection among HRH, thus preventing and mitigating mental health disorders and reinforcing training. These measures were accompanied by specific economic incentives in some of the countries in both sub-regions, such as extra or regular bonuses (see Table 6) and higher remuneration levels. Some countries in South America implemented non-financial measures, such as life insurance or the recognition of COVID-19 as an occupational disease for HRH [12, 13].

**Table 6:** Measures to protect and safeguard HRH

Factor addressed	Measure	Challenges and lessons learned
Prevent, identify and manage HRH infection	Guarantee the availability of PPE	Lack of funding was addressed by reallocating government budget and through donations. Bolivia and Ecuador also received loans Countries made procurement regulations more flexible to facilitate purchasing Governments determined which entities were responsible for guaranteeing PPE distribution Chile established guidelines to promote the rational use of PPE to address shortages
	Provide HRH testing	Testing process varied among countries, depending on funding source for tests, availability of inputs to perform and process tests, and defining institutions to guarantee testing Jamaica performed risk assessment on HRH, supporting testing as needed, according to the results of the assessment
	Identify risk groups by age, comorbidity, pregnancy and childcare who discontinued their activities (South America)	Risk groups impacted HRH availability
	Prioritize HRH in the first phase of vaccination plan	Estimating total number of HRH for vaccination efforts Conduct training among HRH to address concerns about vaccination safety and occurrence of adverse effects
Reduce infection risk Address HRH uncertainty on new pathology	Train HRH on prevention, control, diagnosis and treatment of COVID-19	Technological resources facilitated access to training but did not allow for interaction with the instructor Need to implement mechanisms evaluating the competencies acquired and recognize this learning to advance HRH’s professional career Requires joint action with academia and with the ministries of health and education Need for continuous education/training and pandemic preparedness [22]
Address HRH uncertainty on new pathology and the uncertainty of exposing their family to COVID-19 Improve working conditions	Provide life insurance for HRH (Bolivia, Chile and Peru)	In Bolivia, life insurance covered HRH working in both the public and private sectors. In Peru and Chile, it only covered the public sector
	Recognize COVID-19 as an occupational disease for HRH (Chile and Colombia)	Required coordination between ministries of health and labour was recognized under safety and occupational law
Reinforce HRH mental health	Implement or strengthen plans and guidelines to prevent and mitigate mental health disorders in HRH and their immediate family (South America and Grenada)	There is no information available on the effect of these measures on HRH mental health.^a^
Improve working conditions	Bonuses and pay increases	Need to evaluate which incentives should continue, as well as their design (with possible risks and incentives) and funding sources Need to design a combination of retention mechanisms to address such challenges as distance to work, career growth opportunities and training availability, among others

Elaboration based on PAHO [13] and WHO [12]

^a^Cheng et al. [23] shows the importance of evidence-based interventions providing mental health support to HRH

Factors related to occupational safety also limited the supply of health workers [24]. These included a lack or improper use of personal protective equipment (PPE), non-compliance with infection prevention and control (IPC) protocols [25], mental health disorders [26–28] and insufficient training. Other employment-related factors include work contracts with inadequate and/or late payment without insurance coverage to mitigate the risk of infection or death. Addressing these factors has been a fundamental component of the pandemic response in all the countries studied. Table 6 summarizes these measures, as well as the lessons learned.

### Monitoring, reviewing, and updating the response

Countries moved towards three goals in the process of monitoring, reviewing, and updating the response: (i) strengthening response through primary health care; (ii) planning the workforce needed to implement the vaccination plan; and (iii) securing long-term HRH availability.

In the face of the second wave, countries updated their COVID-19 response plan, placing greater emphasis on care for suspected, probable and confirmed cases through the primary health care system. Moreover, designing the national vaccination plans required reviewing the HRH teams needed to implement it. Chile had the necessary HRH because nursing personnel had already been involved in the national immunization plan, which had approved midwives and dentists to administer vaccines. All other countries needed to utilize mechanisms that would ensure a sufficient supply of vaccinators: in Latin America, this was done through training; and in the Caribbean, through volunteers and retired nurses.

The pandemic has demonstrated the importance of ensuring improved working conditions in the health sector. Balancing decent work with flexibility in hiring and working conditions seems to be the main challenge to the continued use of the hiring mechanisms implemented. Some countries are finding ways to absorb new HRH in a permanent manner and/or to improve contract stability [29]. For example, Ecuador enacted a law through which HRH involved in COVID-19 care under short-term contracts would be hired under a regular contract.

In some countries, HRH were showing hesitancy to COVID-19 vaccination. A study conducted by PAHO in 14 countries of the Caribbean identified that 23% of respondents displayed some level of vaccine hesitancy, chiefly nurses (34%) [30]. There were significant differences among health workers by age (with younger age groups being more hesitant), as well as by categories and specialties. Findings from the study guided interventions to promote vaccine acceptance among health workers, including communication strategies targeting specific HRH categories and age groups. These findings contributed to informed policy development through the HRH Action Task Force and the ministers of health from the CARICOM countries.

A 2022 survey among health workers in 16 countries of Latin America indicated vaccine hesitancy among approximately 3% of all health workers (PAHO, [13]. In 2021, Bolivia conducted a survey among HRH while developing their vaccination plan. It reported that 55% of the health workers surveyed expressed willingness to accept vaccination if the vaccine were available, and 57% would recommend it to a family member. These low acceptance rates for the COVID-19 vaccine put the vaccination plan at risk and provided evidence for the government to implement training sessions to mitigate vaccine hesitancy [31]. A year later, PAHO identified vaccine hesitancy among health workers in Bolivia to have dropped to 2% [13].

## Lessons learned and challenges for future actions and preparedness policies

A key lesson learned in this study is that health workforce readiness is a critical element for preparedness during any health emergency, and that investing in the primary health care workforce is an investment in health security. This paper has identified the importance of HRH data and information analysis, HRH governance and the need to improve the design of effective retention mechanisms through improved working conditions.

Preparing and implementing the COVID-19 response required data and information on HRH, which helped strengthen existing HRH data for information analysis or created mechanisms to collect it. Based on the case studies, we identified three main areas for improving HRH information and its use in decision-making: (1) HRIS; (2) methodologies to estimate HRH needs; and (3) analysis teams to analyse information for informed decision-making.

Through policy dialogue, we identified various opportunities to improve HRIS. The first was the need to adopt standard definitions. The second was to integrate HRIS to combine HRH information from both the public and private sectors, along with national and regional information. For example, policy dialogue in the Caribbean through the HRH Action Task Force identified and chose WHO’s NHWA mechanism for integrating HRH data. Some 34 core HRH indicators were identified and classified into three levels of complexity. This shows how the countries made efforts to identify HRH needs. Some countries used methodologies that they adapted to their own needs, while others used the PAHO model or produced new methodologies. Defining adequate variables and assumptions was important for modelling. An outcome of the policy dialogues is that both the Caribbean and Latin America countries need to develop technology platforms to reinforce the analysis capabilities of their multidisciplinary teams.

Countries coordinated with different stakeholders to align objectives, allocate resources and agree on policy actions. In the Caribbean, ministries of health from the CARICOM countries adopted policy actions to strengthen HRH response and increase vaccine acceptance among health workers there [13]. In Latin America, some countries created a different wage scale for the sub-regions to address the uneven distribution of HRH at the sub-national level. However, this policy proved insufficient to incentivize HRH mobility [12].

The policy dialogues have spurred further evidence being collected for analysis of effective incentives to mobilize HRH. WHO developed the *Working for health 2022–2030 action plan* [32] to strengthen health systems for universal health coverage, while particularly focusing on essential public health functions including emergency preparedness and response. The plan provides a set of strategic actions and a platform for enabling domestic, multisectoral and international cooperation and coordination. Moreover, it proposes concerted actions across three areas: (a) planning and financing, (b) education and employment, and (c) protection and performance.

Policy dialogues that use information and lessons learned contribute to aligning policy priorities and objectives regarding the protection and care of HRH throughout the region. This process can be guided by the *Global health and care worker compact* to rapidly assess, review and monitor good practices.

The pandemic highlighted the importance of protecting HRH’s mental health, with actions centred on prevention. However, policy dialogues have stated a further need to measure the effects of these preventive strategies and move on to developing routine treatment mechanisms for health workers. Additionally, the *World Innovation Summit for Health* reaffirms the obligation to protect and safeguard HRH, by ensuring decent work and a safe, enabling work environment [21]. The lessons we have learned from the Latin American countries suggest the need to update competencies at both the individual and team level, through continuous health education plans within the framework of new technologies.

The pandemic has emphasized the need for countries to be more proactive in their approach to HRH. Challenges ahead include developing legal structures to provide permanent support for the mechanisms created and strengthening those already institutionalized, as well as absorbing the newly recruited HRH to reduce pre-pandemic gaps and improve working conditions. Increasing HRH absorption capacity depends on identifying funding sources and finding ways to accelerate hiring processes.

## Sources included in the case study

- Belize, Ministry of Health (2014). Belize Health Sector Strategic Plan 2014–2024. Available from: https://extranet.who.int/nutrition/gina/sites/default/filesstore/BLZ%202014%20Health%20Sector%20Strategic%20Plan%202014-2024.pdf (accessed 13 Dec 2022).
- Belize, Ministry of Health (2019). Belize Human Resources for Universal Health Strategic Plan 2019–2024. Available from: http://sisco.copolad.eu/web/uploads/documentos/Belize_Health_Sector_Strategic_Plan_2014-2024-April_2014.pdf (accessed 13 Dec 2022).
- Belize, Ministry of Health (2020). Response to the PAHO/WHO questionnaire. Not available on Internet.
- Belize, Ministry of Health (2021). Belize COVID-19 vaccine introduction plan. Not available on Internet.
- Bolivia, Estado Plurinacional de, Asamblea Legislativa Plurinacional (2021 17 Feb). Law No. 1359 of 17 February 2021: Health Emergency Law [Ley N^o^ 1359 del 17 de febrero de 2021: Ley de emergencia sanitaria]. Available from: https://siip.produccion.gob.bo/repSIIP2/files/normativa_12345_180220210e7e.pdf (accessed 15 Dec 2022).
- Bolivia, Estado Plurinacional de, Ministerio de Gobernación (2020). Supreme Decree 4217 of 2020: To authorize contracting of insurance for health personnel related to COVID-19 attention [Decreto supremo N^o^ 4217 del 14 de abril de 2020: Autoriza la contratación de un seguro para los profesionales y trabajadores en salud relacionados con el Coronavirus (COVID-19)]. Available from: https://www.derechoteca.com/gacetabolivia/decreto-supremo-no-4217-del-14-de-abril-de-2020/ (accessed 13 Dec 2020).
- Bolivia, Estado Plurinacional de, Ministerio de Salud y Deportes (2020a). Community surveillance strategy: Plan for containment, mitigation and post-lockdown recovery in addressing COVID-19 [Estrategia de vigilancia comunitaria activa: Plan de contención, mitigación y recuperación post-confinamiento en respuesta a la COVID-19]. Available at: https://www.minsalud.gob.bo/component/jdownloads/?task=download.send&id=550&catid=30&m=0&Itemid=646 (accessed 13 Dec 2022).
- Bolivia, Estado Plurinacional de, Ministerio de Salud y Deportes (2020b). Instructivo MS/DPCH/IN/44/2020, Resolution 245 of 2020: The Minister of Health, Dr. María Eidy Roca de Sangüeza, order obligatory to Ministerial Resolution 245 of 2020, which in Article II resolves to “Authorize the implementation and application of the Integrated Epidemiologic Surveillance System (SIVE) to centralize information about suspected and confirmed COVID-19 cases at a national level” [Instructivo MS/DPCH/IN/44/2020, Resolución 245 of 2020: La Ministra de Salud, Dra. María Eidy Roca de, instruye dar cumplimiento de forma obligatoria a la Resolución Ministerial No. 0245 de 4 de mayo de 2020, misma que a través de su artículo segundo resuelve “Autorizar la implementación y aplicación del Sistema Integrado de Vigilancia Epidemiológica (SIVE), para centralizar la información de casos sospechosos y confirmados de COVID-19 a nivel nacional, en todos los niveles de gestión de información, así como los laboratorios del Sistema Público y Privado de Salud, reconocidas por el Ministerio de Salud”]. Available from: https://www.minsalud.gob.bo/programas-de-salud/coordinacion-nacional-de-laboratorios/8-institucional/4442-todas-las-comunicaciones-oficiales-del-ministerio-de-salud-relacionado-al-covid-19-gestion-2020 (conduct internal search for “resolución 245 de 2020” for direct link; accessed 13 Dec 2022).
- Bolivia, Estado Plurinacional de, Ministerio de Salud y Deportes (2021a). Report of COVID-19 infections in Bolivia No. 394, 2021 (Reporte de contagios COVID-19 en Bolivia N^o^ 394, 2021). Available from: https://www.unidoscontraelcovid.gob.bo/index.php/2021/04/14/reporte-de-contagios-covid-19-en-bolivia-n394 (access from site owner required).
- Bolivia, Estado Plurinacional de, Ministerio de Salud y Deportes (2021b). Instructive MSyD/VPVEyMT/DGE/UPyCE/PAI/IN/38/2021: All health personnel carrying out vaccination against COVID-19 in the national territory is instructed to comply with technical norms … [Instructive MSyD/VPVEyMT/DGE/UPyCE/PAI/IN/38/2021: Se instruye a todo el personal de salud que realiza la vacunación contra la COVID-19 en todo el territorio nacional, dar cumplimiento a la norma técnica …]. Available from: https://www.minsalud.gob.bo/component/jdownloads/?task=download.send&id=605&catid=30&m=0&Itemid=646 (conduct internal search for “plan de vacunación 2021,” for direct link; accessed 13 Dec 2022).
- Chile, Congreso Nacional (2020 31 Dec). Law 21306 to grant compensation readjustment to public sector workers, to grant indicated bonuses and other benefits and to modify various legal bodies (Ministry of Housing) [Ley 21306: otorga reajuste de remuneraciones a los trabajadores del sector público, concede aguinaldos que señala, concede otros beneficios que indica, y modifica diversos cuerpos legales (Ministerio de Hacienda)]. Available from: https://www.bcn.cl/leychile/navegar?idNorma=1154076 (accessed 15 Dec 2022).
- Chile, Ministerio de Ciencia, Tecnología, Conocimiento e Innovación (2020). A look at data culture in Chile: Challenges and lessons from the COVID-19 pandemic [Una mirada a la cultura de datos en Chile: Desafíos y lecciones de la pandemia por COVID-19]. Available from: https://www.minciencia.gob.cl/legacy-files/whitepaper_datos_covid19.pdf (accessed 13 Dec 2022).
- Chile, Ministerio de Salud (2015). Decree 90 of 2015: To approve the regulations for the exercise of the auxiliary professions of Medicine, Dentistry, Chemistry, Pharmacy and others [Decreto 90: Aprueba reglamento para el ejercicio de las profesiones auxiliares de la medicina, odontología, química y farmacia y otras, y deroga Decretos Nº 261, de 1978, y Nº 1.704, de 1993, ambos del Ministerio de Salud). Available from: https://www.bcn.cl/leychile/navegar?idNorma=1099220 (accessed 14 Dec 2022).
- Chile, Ministerio de Salud (2017). Gaps in health workforce by health service and specialist area. Glosa 01, letter i. Budget Law 2017 [Informe sobre brechas de personal de salud por servicio de salud, glosa 01, letra i, Ley de Presupuestos año 2017]. Available from: https://www.minsal.cl/wp-content/uploads/2015/08/Informe-Brechas-RHS-en-Sector-P%C3%BAblico_Abril2017.pdf (accessed 14 Dec 2022).
- Chile, Ministerio de Salud (2020a). Epidemiological report: Characteristics of health workers with confirmed COVID-19 [Informe epidemiológico: Características del personal de salud con casos confirmados de COVID-19, Chile: Semanas epidemiológicas 10 a la 37, año 2020]. Available from: https://www.minsal.cl/wp-content/uploads/2020/09/Personal-de-Salud-Covid-3092020.pdf (accessed 14 Dec 2022).
- Chile, Ministerio de Salud (2020b). Coordination protocol for epidemiological surveillance actions during the COVID-19 pandemic in Chile: National Strategy for Testing, Traceability and Isolation [Protocolo de coordinación para acciones de vigilancia epidemiológica durante la pandemia COVID-19 en Chile: Estrategia nacional de testeo, trazabilidad y aislamiento. Available from: https://www.minsal.cl/wp-content/uploads/2020/07/Estrategia-Testeo-Trazabilidad-y-Aislamiento.pdf (accessed 14 Dec 2022).
- Chile, Ministerio de Salud (2020c, 19 Mar). Order 718 of 2020: To give instructions within the Health Alert for the COVID-19 coronavirus outbreak framework [Orden 718 de 2020: Imparte instrucciones en al marco de Alerta Sanitaria por brote de coronavirus COVID-19]. Available from: http://www.sirh.cl/sites/default/files/biblioteca/ord._n_718_del_20.03.2020_imparte_instrucciones_en_el_marco_de_alerta_sanitaria_por_brote_de_coronavirus_covid_19_003.pdf (accessed 14 Dec 2022).
- Chile, Ministerio de Salud (2020d). Chile's experience. The response to COVID-19 from Human Resources for Health. PAHO's Webinar [Experiencia de Chile: la respuesta al COVID-19 desde los Recursos Humanos de Salud]. Available from: https://www.observatoriorh.org/sites/default/files/webfiles/fulltext/2020/web_sur_jul/3_chile.pdf (accessed 14 Dec 2022).
- Chile, Ministerio de Salud (2020e). President Piñera announces the enactment of a bonus for health personnel due to the COVID-19 pandemic [Presidente Piñera anuncia promulgación de bono para personal de la salud por pandemia de COVID-19]. Available from: https://www.minsal.cl/presidente-pinera-anuncia-promulgacion-de-bono-para-personal-de-la-salud-por-pandemia-de-covid-19/ (accessed 14 Dec 2022).
- Chile, Ministerio de Salud (2020f). Circular C37 N° 2 of 2020: To complement the correct use of Personal Protective Equipment in the context of the COVID-19 pandemic [Circular C37 N^o^ 2 de 2020: Racionalización del uso de equipos de protección personal (EPPE) en el contexto de la atención para pacientes durante la pandemia de COVID-19]. Available from: https://www.minsal.cl/wp-content/uploads/2020/05/03-abr-Circular-2-Racionalizacion-uso-EPP-en-contexto-atencion-pacientes-durante-pandemia.pdf (accessed 14 Dec 2022).
- Chile, Ministerio de Salud (2020 g 10 May). Ministry of Health highlights start of rapid tests in health officials [Ministerio de Salud destaca inicio de testeos rápidos en funcionarios de salud]. Available from: https://www.minsal.cl/ministerio-de-salud-destaca-inicio-de-testeos-rapidos-en-funcionarios-de-salud/ (accessed 14 Dec 2022).
- Chile, Ministerio de Salud (2020 h 17 Mar). Resolution 182 of 2020: To establish an indicated work modality for officials of the divisions of the Ministry of Health, Cabinets and Health Seremis, within the COVID-19 outbreak framework [Resolución 182 de 2020: Establece modalidad de trabajo que indica, para funcionarios y funcionarias de las divisiones del Ministerio de Salud, en el marco del brote de COVID-19]. Available from: https://www.colegiofarmaceutico.cl/index.php/noticias-nacionales/coronavirus-informate/informacion-del-gobierno/3484-resolucion-exenta-182-17-de-marzo-de-2020 (accessed 14 Dec 2022).
- Chile, Ministerio de Salud (2020i, 1 Jun). Health authorities sign agreement for the delivery of insurance to workers due to COVID-19 [Autoridades de Salud suscriben acuerdo para la entrega de seguro a trabajadores por COVID-19]. Available from: https://www.minsal.cl/autoridades-de-salud-suscriben-acuerdo-para-la-entrega-de-seguro-a-trabajadores-por-covid-19/ (accessed 14 Dec 2022).
- Chile, Ministerio de Salud (2021a). Gaps in health workforce by health service in the public network: situation by health service, departments and medical specialties: Glosa 01c, Budget Law 2020, June 2021 [Brechas de personal de salud red asistencial pública: Situación por Servicio de Salud, Plantas y Especialidades Médicas. Glosa N° 01 c—Ley de Presupuesto N° 21.289, junio de 2021]. Available from: https://www.minsal.cl/wp-content/uploads/2021/07/Informe-de-Glosa-01c_Informe-Brechas-Junio-2021.pdf (accessed 14 Dec 2022).
- Chile, Ministerio de Salud (2021b). Epidemiological report: Characteristics of health workers with confirmed COVID-19 [Informe epidemiológico: Características del personal de salud con casos confirmados de COVID-19, Chile: Semanas epidemiológicas 10 a la 53, año 2020]. Available from: https://www.minsal.cl/wp-content/uploads/2021/02/2021-01-21_Informe-PS-COVID-19.pdf (accessed 14 Dec 2022).
- Chile, Ministerio de Salud (2021c, 10 Feb). Resolution 136 of 2021: To complement Ministry of Health Resolution 1138 of 2020, that approves technical and operational guidelines for SARS COV-2] [Extenta 136 de 2021: Complementa Resolución Extenta 1138, de 2020, que aprueba lineamientos técnico operativos vacunación SARS COV-2]. Available from: https://www.minsal.cl/wp-content/uploads/2021/02/RES.-EXENTA-N-136_.pdf (accessed 14 Dec 2022).
- Chile, Ministerio de Salud (n.d.) Human Resources Information System [Sistema de Información de Recursos Humanos/SIRH]. Available from: http://www.sirh.cl/que-es-sirh (accessed 14 Dec 2022).
- Chile, Ministerio de Salud. Chile Attends: AUGE-GES Plan (2 Aug 2021) [Chile atiende: Plan AUGE-GES (2 agosto 2021)]. Available from: https://www.chileatiende.gob.cl/fichas/2464-plan-auge-ges (accessed 13 Dec 2022).
- Chile, Superintendencia de Salud (2013). Health professionals and technicians must be registered in the Registry of Individual Providers in order to grant AUGE care [Los profesionales y técnicos de salud deben ser registrados en el Registro de Proveedores Individuales para recibir cuidados de AUGE]. Available from: https://diprece.minsal.cl/le-informamos/auge/normativas-auge (accessed 27 Dec 2022)/http://www.supersalud.gob.cl/portal/w3-article-8127.html (access to main site not always available).
- Chile, Superintendencia del Seguro Social (27 Apr 2020). Dictum 1482 of 2020: To provide instructions regarding the origin of the COVID-19 disease qualifications on health personnel of health facilities and those that have been determined as close contacts [Dictamen 1482 of 2020: Imparte instrucciones respecto a la calificación del origen de la enfermedad COVID-19 que afecte al personal de establecimientos de salud y aquellos que han sido determinados como contactos estrechos]. Available from: https://www.suseso.cl/612/w3-article-589773.html (accessed 15 Dec 2022).
- Colombia, Instituto Nacional de Salud (2020, 2021a). COVID-19 in the health workforce in Colombia [COVID-19 en personal de la salud en Colombia]. Available from: https://www.ins.gov.co/Noticias/Paginas/coronavirus-personal-salud.aspx (accessed 15 Dec 2022).
- Colombia, Instituto Nacional de Salud (2021b). COVID-19 in Colombia [COVID-19 en Colombia].https://www.ins.gov.co/Noticias/Paginas/coronavirus-casos.aspx (accessed 15 Dec 2022).
- Colombia, Ministerio de Salud y Protección Social (2020a). Estimations of availability, requirements and gaps in Human Talent for Health for COVID-19 attention in Intensive Care Units, Intermediate Care and Hospitalization of low complexity [Estimaciones de disponibilidad, requerimientos y Brechas de Talento Humano en Salud –THS– para la atención COVID-19 en Unidades de Cuidado Intensivo, Cuidado Intermedios y Hospitalizacion de baja complejidad]. Available from: https://www.minsalud.gov.co/sites/rid/Lists/BibliotecaDigital/RIDE/VS/TH/estimaciones-thcovid19.pdf (accessed 14 Dec 2022).
- Colombia, Ministerio de Salud y Protección Social (2020b). National Government and Ascofame designed a multidisciplinary course for attention of COVID-19 patients [Gobierno Nacional y Ascofame diseñaron curso multidisciplinario para manejo de pacientes covid-19]. Available from: https://www.minsalud.gov.co/Paginas/Gobierno-Nacional-y-Ascofame-disenaron-curso-multidisciplinario-para-manejo-de-pacientes-covid-19--.aspx (accessed 14 Dec 2022).
- Colombia, Ministerio de Salud y Protección Social (2020c). Action Plan for the Provision of Health Services during the Containment and Mitigation Stages of COVID-19 [Plan de acción para la prestación de servicios de salud durante las etapas de contención y mitigación de la pandemia por SARS-Cov-2 (COVID-19)]. Available from: https://www.minsalud.gov.co/Ministerio/Institucional/Procesos%20y%20procedimientos/PSSS01.pdf (accessed 14 Dec 2022).
- Colombia, Ministerio de Salud y Protección Social (2020d, 15 Oct). Recognition of human talent in health is a reality. Press release No. 833 [El reconocimiento del talento humano en salud es una realidad. Boletín de Prensa No. 833 de 2020]. Available from: https://www.minsalud.gov.co/Paginas/El-reconocimiento-al-talento-humano-en-salud-es-una-realidad.aspx (accessed 14 Dec 2022).
- Colombia, Ministerio de Salud y Protección Social (2020e). Resolution 1774 of 2020: To define occupational profiles for one-time economic recognition in favour of human talent in health that provides services to suspected or diagnosed COVID-19 patients, the methodology for calculating the amount and the mechanism of payment by the Administrator of the General System of Social Security in Health Resources – ADRES [Ministerio de Salud y Protección Social Resolución Número 1774 del 6 de octubre de 2020: Por la cual se definen los perfiles ocupacionales para el reconocimiento económico por una única vez en favor del talento humano en salud que preste sus servicios a pacientes con sospecha o diagnóstico de Coronavirus COVID -19, la metodología para el cálculo del monto, y el mecanismo de giro por parte de la Administradora de los Recursos del Sistema General de Seguridad Social en Salud—ADRES]. Available from: https://www.minsalud.gov.co/sites/rid/Lists/BibliotecaDigital/RIDE/DE/DIJ/resolucion-1774-de-2020.pdf (accessed 14 Dec 2022).
- Colombia, Ministerio de Salud y Protección Social (2020f 19 May). Resolution 779 of 2020: To formalize health response strategy adopted to face the SARS CoV-2 (COVID-19) pandemic in Colombia and to create an advisory committee to guide policy decisions in relation to the pandemic [Resolución 779 de 2020: Por la cual se formaliza la estrategia de respuesta sanitaria adoptada para enfrentar la pandemia de SARS CoV-2 (COVID-19) en Colombia y se crea un comité asesor para orientar las decisiones de política en relación con la pandemia]. Available from: (https://www.minsalud.gov.co/Normatividad_Nuevo/Resoluci%C3%B3n%20No.%20779%20de%202020.pdf (accessed 14 Dec 2022).
- Colombia, Ministerio de Salud y Protección Social (2021 25 Feb). Health provider institutions must strengthen their vaccination network: Press release No. 294 [IPS deben fortalecer su red de vacunación: Boletín de Prensa 294 de 2021]. Available from: https://www.minsalud.gov.co/Paginas/IPS-deben-fortalecer-su-red-de-vacunacion.aspx (accessed 14 Dec 2022).
- Colombia, Ministerio de Salud y Protección Social/Observatorio para el Talento Humano en Salud (2017). Follow-up Indicators [Indicadores de seguimiento]. Available from: https://www.sispro.gov.co/observatorios/ontalentohumano/Paginas/Indicadores.aspx (accessed 20 Dec 2022).
- Colombia, Ministerio de Trabajo (2020). Decree 676 of 2020: COVID-19 is included as a work-related disease [Decreto 676 de 2020: Se incluye el COVID-19 como enfermedad laboral]. Available from: https://acmineria.com.co/normativa/decreto-676-de-2020-se-incluye-el-covid-19-como-enfermedad-laboral/ (accessed 13 Dec 2022).
- Colombia, Presidencia de la República (2020). Legislative Decree 538 of 2020 through which an economic, social and ecological state of emergency is declared throughout the national territory [Decreto Legislativo 538 de 2020 por el cual se declara un estado de emergencia económica, social y ecológica en todo el territorio nacional]. Available from: https://www.funcionpublica.gov.co/eva/gestornormativo/norma.php?i=111934 (accessed 15 Dec 2022).
- Ecuador, Instituto Nacional de Estadísticas y Censo (2020) Technical Bulletin N°-01–2020-RAS: Statistical Register of Health Resources and Activities—RAS 2018 [Registro estadístico de recursos y actividad de salud—RAS 2018]. Available from: https://www.ecuadorencifras.gob.ec/documentos/web-inec/Estadisticas_Sociales/Recursos_Actividades_de_Salud/RAS_2018/Boletin_Tecnico_RAS_2018.pdf (accessed 15 Dec 2022).
- Ecuador, Ministerio de Salud Pública (2020). The Ministry of Public Health optimizes resources to hire 2850 health professionals [El MSP optimiza recursos para contratar a 2.850 profesionales de salud]. Available from: https://www.salud.gob.ec/el-msp-optimiza-recursos-para-contratar-a-2-850-profesionales-de-salud/ (accessed 14 Dec 2022).
- Ecuador, Ministerio de Salud Pública (2021b). COVID-19 Vaccination Plan: Ecuador 2020–2021 [Plan de trabajo vacunación COVID-19: Ecuador, 2020–2021]. Available from: https://www.salud.gob.ec/wp-content/uploads/2021/03/DOCUMENTO-PLAN-DE-VACUNACIO%CC%81N-ECUADOR-VS-FINAL_r.pdf (accessed 14 Dec 2022).
- Ecuador, Ministerio de Salud Pública/Dirección Nacional de Vigilancia Epidemiológica (2021a). Behaviour of COVID-19 in Ecuador [Comportamiento del COVID-19 en Ecuador]. Available from: https://www.salud.gob.ec/coronavirus-covid19-ecuador/ (accessed 20 Dec 2022).
- Ecuador, Portal único de trámites ciudadanos [Citizen Procedures Single Portal] (gob.ec) (2022). Quality Assurance Agency for Health and Medical Services and Prepaid Medicine [Agencia de Aseguramiento de la Calidad de los Servicios de Salud y Medicina prepagada ACESS]. Available from: https://www.gob.ec/acess/tramites/atencion-registro-titulos-profesionales-salud-nacionales-extranjeros-ciencias-salud (accessed 13 Dec 2022).
- Ecuador, Presidencia Constitucional. General Regulation of the Humanitarian Support Law: Executive Decree; 1165, 5 Oct 2020 [Reglamento general de la ley orgánica de apoyo humanitario: Decreto Ejecutivo 1165, 5 oct 2020]. Available from: https://www.igualdad.gob.ec/wp-content/uploads/downloads/2020/10/reglamento_general_ley_organica_apoyo_humanitario_oct2020.pdf (accessed 13 Dec 2022).
- Grenada, Ministry of Health (2009). Grenada National Influenza Pandemic Preparedness Plan, Operation Manual. Available from: https://www3.paho.org/hq/images/stories/AD/HSD/CD/INFLUENZA/grenada%20nippp.pdf (accessed 14 Dec 2022).
- Grenada, Ministry of Health (2015). Strategic Plan for Health 2016–2025. Available from: https://gov.gd/sites/moh/files/grenada_health_sector_strategic_plan_2016-2015_approved.pdf (accessed 14 Dec 2022).
- Grenada, Ministry of Health (2020). Terms of Reference, Environmental Health Warden Programme. Not available on Internet.
- Grenada, Ministry of Health (2021). National Deployment and Vaccination Plan for COVID-19 Vaccines. Not available on Internet.
- Jamaica, Ministry of Health (2019). Vision for health 2030. Ten-year strategic plan 2019–2030. Available from: https://www.moh.gov.jm/data/vision-for-health-2030-ten-year-strategic-plan-2019-2030/ (accessed 14 Dec 2020).
- Jamaica, Ministry of Health (2021). Interim vaccination logistics from the vial to the arm. Available from: https://www.moh.gov.jm/wp-content/uploads/2021/03/MOHW-Interim-Vaccination-Implementation-Plan-02.03.2021-Final.pdf (accessed 14 Dec 2022).
- Migration Policy Institute (MPI). Migration Data Hub. Washington, DC: 2021. Available from: https://www.migrationpolicy.org/programs/migration-data-hub (accessed 13 Dec 2022).
- Pan American Health Organization/World Health Organization (PAHO/WHO). Health in the Americas. Summary: Regional Outlook and Country Profiles. Washington, DC: PAHO/WHO; 2017. Available from: https://iris.paho.org/handle/10665.2/34321 (accessed 15 Dec 2022).
- Pan American Health Organization/World Health Organization (PAHO/WHO). Health Workers’ Perception and Migration in the Caribbean Region. Washington, DC: PAHO/WHO; 2019. Available from: https://www.paho.org/en/documents/health-workers-perception-and-migration-caribbean-region (accessed 15 Dec 2022).
- Pan American Health Organization/World Health Organization (PAHO/WHO). Human Resources for Health and the COVID-19 Response in the Caribbean. Washington, DC: PAHO/WHO; 2020. Available from: https://www.paho.org/en/documents/human-resources-health-and-covid-19-response-caribbean (accessed 15 Dec 2022).
- Pan American Health Organization/World Health Organization (PAHO/WHO). Concerns, attitudes, and intended practices of healthcare workers toward COVID-19 vaccination in the Caribbean. Washington, DC; PAHO/WHO; 2021a. Available from: https://iris.paho.org/handle/10665.2/54964 (accessed 15 Dec 2022).
- Pan American Health Organization/World Health Organization (PAHO/WHO). Health in the Americas. Summary: regional outlook and country profiles. Washington, DC; PAHO/WHO; 2017. Available from: https://iris.paho.org/handle/10665.2/34321 (accessed 15 Dec 2022).
- Pan American Health Organization/World Health Organization (PAHO/WHO). PAHO/WHO donates personal protective equipment (PPEs) and biomedical equipment to support the Karl Heusner Memorial Hospital. Belize City: PAHO/WHO Country Office; 2021d 10 May. Available from: https://www.paho.org/en/news/10-5-2021-pahowho-donate-personal-protective-equipment-ppes-and-biomedical-equipment-support (accessed 15 Dec 2022).
- Pan American Health Organization/World Health Organization (PAHO/WHO). Policy Brief: Addressing COVID-19 vaccine hesitancy among healthcare workers in the Caribbean. Washington, DC: PAHO; 2021e. Available from: https://www.paho.org/en/documents/policy-brief-addressing-covid-19-vaccine-hesitancy-among-healthcare-workers-caribbean (accessed 15 Dec 2022).
- Pan American Health Organization/World Health Organization (PAHO/WHO). Impact of COVID-19 on human resources for health and policy response: the case of Belize, Grenada and Jamaica. Overview of findings from three Caribbean countries. Washington, DC: [PAHO/WHO; 2022]. Available from: https://iris.paho.org/bitstream/handle/10665.2/56262/PAHOCRBCOVID19220001_eng.pdf?sequence=1&isAllowed=y (accessed 15 Dec 2022).
- Pan American Health Organization/World Health Organization (PAHO/WHO). Impacto de la COVID-19 en los recursos humanos para la salud y respuesta de política: el caso del Estado Plurinacional de Bolivia, Chile, Colombia, Ecuador y el Perú: síntesis de hallazgos en cinco países de América Latina Washington, DC: PAHO/WHO; 2023. Available from: https://www.who.int/es/publications/i/item/9789240039001 (accessed 15 Dec 2022)
- Pan American Health Organization/World Health Organization (PAHO/WHO). El impacto de la COVID-19 en los recursos humanos para la salud y respuestas de política El caso de cinco países de América Latina. Washington, DC: PAHO/WHO; forthcoming 2023.
- Pan American Health Organization/World Health Organization (PAHO/WHO). Epidemiological update: coronavirus disease (COVID-19). Washington, DC: PAHO/WHO; 2021 14 Apr. Available from: https://www.paho.org/es/documentos/actualizacion-epidemiologica-enfermedad-por-coronavirus-covid-19-14-abril-2021 (accessed 15 Dec 2022).
- Peru, Ministerio de Economía y Finanzas (2020). Urgency Decree 032–2020: To dictate complementary measures for the Health Sector in the Health Emergency due to the effects of COVID-19 framework [Decreto de urgencia No 032–2020: Decreto de urgencia que dicta medidas extraordinarias destinadas a garantizar la respuesta sanitaria para la atención de la emergencia producida por el COVID-19]. El Peruano: 2020; XXXVII (15,331): 23 de marzo de 2020]. Available from: https://cdn.www.gob.pe/uploads/document/file/571634/DU032_2020.pdf (accessed 13 Dec 2022).
- Peru, Ministerio de Salud (2019). Statistical Compendium: Information on Human Resources for Health: Peru 2013–2018 [Compendio Estadístico: Información de Recursos Humanos del Sector Salud, Perú, 2013–2018]. Available from: http://bvs.minsa.gob.pe/local/MINSA/10896.pdf (accessed 15 Dec 2022).
- Peru, Ministerio de Salud (2020b 6 Sep). MoH-Peru will install 17 CRATs in nine districts of the northern area of Lima for testing and attention of COVID-19: Press Release [Minsa instalará 17 CRAT en nueve distritos de la zona norte de Lima para el descarte y atención de COVID-19: Nota de prensa]. Available from: https://www.gob.pe/institucion/minsa/noticias/300647-minsa-instalara-17-crat-en-nueve-distritos-de-la-zona-norte-de-lima-para-el-descarte-y-atencion-de-covid-19 (accessed 15 Dec 2022).
- Peru, Ministerio de Salud (2020c). Supreme Decree 010–2020-SA: To approve the Action Plan: Surveillance, containment, and attention of cases of the new COVID-19 in Peru and the list of goods and services required for the activities of the health emergency … [Decreto Supremo que aprueba el plan de acción y la relación de bienes y servicios requeridos para enfrentar la Emergencia Sanitaria …]. Available from: https://cdn.www.gob.pe/uploads/document/file/566437/DS_010-2020-SA.PDF (accessed 15 Dec 2022).
- Peru, Ministerio de Salud (2020d 25 May). Resolution No. 316–2020-MINSA: To create the Surveillance Committee for the Assignment and Use of Personal Protective Equipment in each Health Services Provider Institution [Resolución N^o^ 316–2020-MINSA: Comité de Vigilancia de asignación y uso de Equipos de Protección Personal]. Available from: https://www.gob.pe/institucion/minsa/normas-legales/587393-316-2020-minsa (accessed 15 Dec 2022).
- Peru, Ministerio de Salud (2020e 6 May). Technical document: Attention of people affected by COVID-19 in critical care area—Guide [Documento técnico: Manejo de personas afectadas por COVID-19 en áreas de atención crítica—Guía]. Available from: https://www.gob.pe/institucion/minsa/informes-publicaciones/558862-documento-tecnico-manejo-de-personas-afectadas-por-covid-19-en-areas-de-atencion-critica (accessed 15 Dec 2022).
- Peru, Ministerio de Salud (2020f 20 Oct). Resolution 848–2020-MINSA: COVID-19 National Vaccination Plan [Resolución Ministerial N.° 848–2020-MINSA: Plan Nacional de Vacunación contra la COVID-19]. Available from: https://www.gob.pe/institucion/minsa/normas-legales/1293043-848-2020-minsa (accessed 15 Dec 2022).
- Peru, Ministerio de Salud/Centro Nacional de Epidemiología, Prevención y Control de Enfermedades (2020a). Current situation COVID-19 Peru 2020 [Situación actual “COVID-19” Perú 2020 (31 de agosto)]. Available from: https://www.dge.gob.pe/portal/docs/tools/coronavirus/coronavirus310820.pdf (accessed 15 Dec 2022).
- Peru, Ministerio de Salud/Observatorio de Recursos Humanos para la Salud (2020). Information on Human Resources for Health from the Ministry of Health and Regional Governments [Información de recursos humanos en salud del MINSA y gobiernos regionales, 2020]. Available from: (https://app.powerbi.com/view?r=eyJrIjoiOTE3ZmEwODMtMmFlMy00ZDA4LTg5ZGEtMGMzZDBjNmU2ZTMxIiwidCI6ImI3ZDJiMWZkLWU3NjMtNDY5ZS05NjE5LWM5M2I3MmEyYzUwMyJ9&pageName=ReportSection59d61fb40feb0d4a46db (accessed 15 Dec 2022).
- Peru, Presidencia de la República (2020 15 Mar). Urgency Decree 026–2020: To establish various exceptional and temporary measures to prevent the spread of COVID-19 in the national territory [D.U. 026–2020: Decreto de Urgencia que establece diversas medidas excepcionales y temporales para prevenir la propagación del Coronavirus (COVID-19) en el territorio nacional. Available from: https://cdn.www.gob.pe/uploads/document/file/566447/DU026-20201864948-1.pdf (accessed 15 Dec 2022).
- University of Belize. About UB. Belmopan: University of Belize; 2020. Available from: https://www.ub.edu.bz/about-ub/#ourstory (accessed 15 Dec 2022).
- World Health Organization (WHO) (2021d). National Health Workforce Accounts Data Portal. Geneva: WHO; 2021d. Available from: https://apps.who.int/nhwaportal/Home/Index (accessed 15 Dec 2022).
- World Health Organization (WHO). A combined week sex age group: COVID-19 Database (COVMART Database). Geneva; WHO; 2021a. Available from: https://extranet.who.int/xmart4/NCOV/data/A_COMBINED_WEEK_SEX_AGEGROUP (access only to registered users).
- World Health Organization (WHO). COVID-19 Weekly epidemiological update. Geneva: WHO; 2021b. Available from: https://www.who.int/publications/m/item/weekly-epidemiological-update-on-covid-19---31-march-2021) (accessed 15 Dec 2022).
- World Health Organization (WHO). Impact of COVID-19 on human resources for health and policy response: the case of Plurinational State of Bolivia, Chile, Colombia, Ecuador and Peru. Overview of findings from five Latin American countries. Geneva: WHO; 2021c. Available from: https://apps.who.int/iris/handle/10665/350640 (accessed 15 Dec 2021).

## Data Availability

The data that support the findings of this study are available from “Source included in the case studies” section (mainly the sources included in the case studies: though not cited in this paper, they still function as sources of information).

## Abbreviations

AFRO: Regional Office for Africa of the World Health Organization
CARICOM: Caribbean Community
COVID-19: Corona Virus Disease 2019 (SARS-COV-2)
EPIVIGILIA: Epidemiologic Surveillance System, Chile
HLM: Health Labour Market
HIS: Health information system
HRH: Human resources for health (meaning health workers or the health workforce)
HRIS: Health resources for health information system, also known as HRHIS or Human Resources Information System
ICU: Intensive care unit
INFORHUS: National Registry of Health Personnel Application, Peru
INS: National Health Institute of Colombia
IPC: Infection prevention and control
ITU: Intensive treatment unit
LAC: Latin America and the Caribbean
MoH: Ministry of health
MoH-Belize: Ministry of Health and Wellness of Belize
MoH-Bolivia: Ministry of Health and Sports of the Plurinational State of Bolivia
MoH-Chile: Ministry of Health of Chile
MoH-Colombia: Ministry of Health and Social Protection of Colombia
MoH-Ecuador: Ministry of Public Health of Ecuador
MoH-Grenada: Ministry of Health, Social Security and International Business of Grenada
MoH-Jamaica: Ministry of Health and Wellness of Jamaica
MoH-Peru: Ministry of Health of Peru
NHWA: WHO’s National Health Workforce Accounts
ORAS-CONHU: Andean Health Organism – Hipólito Unanue Agreement *(Organismo Andino de Salud/Acuerdo Hipólito Unanue)*
PAHO: Pan American Health Organization
PPE: Personal protective equipment
RAS: Statistical Registry of Health Resources and Activities, Ecuador
ReTHUS: National Registry of Healthcare Talent, Colombia
RUES: Single Registry of Health Establishments, Bolivia
SIREPRO: Professional Registration System, Bolivia
SIRH: Human Resources Information System, Chile
SIVIGILA: Public Health Surveillance System, Colombia
VIEPI: Epidemiological Surveillance System Ecuador
WHO: World Health Organization
WISM: Workload Indicators of Staffing Need

## Footnotes

1 First, an acute demand on the health system’s capacity and workforce. Second, volume demand for acute care services. Third, attrition in the supply of health workers due to illness, exposure/quarantine, family illness, lack of childcare, and fear, as well as related effects on mental health.

2 Out of 112 countries, 66% reported this as a reason.

3 Mixed analytical methods were used in this working paper to estimate the global probable ranges of HRH deaths due to COVID-19.

4 This also makes them prone to such develop such disorders as burnout or post-traumatic stress disorder (PTSD) [33].

5 These countries are members of the PAHO Sub-regional Programme for the Andean Sub-region and the Andean Health Organisation.

6 These countries are Member States of the PAHO’s Caribbean subregion and CARICOM.

7 These countries expressed both an interest in documenting their COVID-19 response and a willingness to review and discuss it in the context of HRH [12, 16]

8 The NHWA is a mechanism to collate and use a set of standardized indicators to generate reliable HRH information and evidence.

9 WHO has recently defined governance roles. The conceptual framework adopted expresses the idea that governance includes a legal framework. This is supported by a strong stakeholders’ coalition upheld by policy dialogue processes, thus providing a strategic direction to the health system as well as the necessary means to implement policies and capacities to keep governments accountable [34].
